# The Influence of Cross-Linguistic Similarity and Language Background on Writing to Dictation

**DOI:** 10.3389/fpsyg.2021.679956

**Published:** 2021-09-28

**Authors:** Antonio Iniesta, Eleonora Rossi, M. Teresa Bajo, Daniela Paolieri

**Affiliations:** ^1^Department of Experimental Psychology, Mind, Brain and Behavior Research Center (CIMCYC), University of Granada, Granada, Spain; ^2^Department of Linguistics, University of Florida, Gainesville, FL, United States

**Keywords:** bilingual writing, language co-activation, orthographic/phonological similarity, heritage speakers, writing to dictation

## Abstract

This study used a word dictation task to examine the influence of a variety of factors on word writing production: cognate status (cognate vs. non-cognate words), orthographic (OS) and phonological similarity (PS) within the set of cognate words, and language learning background [late bilinguals (LBs) with academic literacy and formal instruction in English and Spanish, and heritage speakers (HSs) with academic literacy and formal instruction only in English]. Both accuracy and reaction times for the first key pressed by participants (indicating lexical access), and the time required to type the rest of the word after the first keypress (indicating sublexical processing) was assessed. The results revealed an effect of PS on the dictation task particularly for the first keypress. That is, cognates with high PS were processed faster than cognates with low PS. In contrast to reading studies in which PS only revealed a significant effect when the OS between languages was high (O+P+ vs. O+P−), in the dictation to writing task, the phonology had a more general effect across all conditions, regardless of the level of OS. On the other hand, OS tended to be more influential for typing the rest of the word. This pattern is interpreted as indicating the importance of phonology (and PS in cognates) for initial lexical retrieval when the input is aural. In addition, the role of OS and PS during co-activation was different between groups probably due to the participants’ linguistic learning environment. Concretely, HSs were found to show relatively lower OS effects, which is attributed to the greater emphasis on spoken language in their Spanish language learning experiences, compared to the formal education received by the LBs. Thus, the study demonstrates that PS can influence lexical processing of cognates, as long as the task demands specifically require phonological processing, and that variations in language learning experiences also modulate lexical processing in bilinguals.

## Introduction

A central question in bilingual research has been to determine how bilinguals manage the use of words from different languages (Kroll et al., 2013; Costa and Sebastián-Gallés, 2014). There is evidence that bilinguals co-activate their two languages, even in single language contexts (e.g., Van Heuven et al., 1998; Van Hell and Dijkstra, 2002; Von Studnitz and Green, 2002; Marian and Spivey, 2003; Hoshino and Kroll, 2008; Macizo, 2016) and that this parallel co-activation may facilitate (Costa et al., 2000; Christoffels et al., 2007; Voga and Grainger, 2007; Lemhöfer et al., 2008) or hinder access to intended words (Gollan et al., 2005; Ivanova and Costa, 2008). Under the assumption that the two languages are co-activated (“non-selective” activation of the two languages; Dijkstra and Van Heuven, 2002), a key question is whether bilingual language co-activation is modulated at different linguistic levels (e.g., lexical, orthographic, and phonological) depending on the linguistic tasks (i.e., reading, speaking, and writing). Critically, one question that is untapped in the literature is how these various levels of co-activation and control thereof vary for different bilingual populations with diverse language experiences.

Orthographic processing has been the focus of most bilingual word recognition studies (e.g., van Heuven et al., 1998; Van Kesteren et al., 2012; Casaponsa et al., 2014; Hoversten et al., 2017). The cross-linguistic influence of the two bilingual orthographic codes has been strongly supported by experimental evidence using cognate words. Cognate words are words that have the same meaning and form representation in two or more languages (e.g., “chocolate” in English is translated as “chocolate” in Spanish). Behavioral studies using different experimental tasks (lexical decision, word recognition, naming, and translation) have demonstrated that cognate words are processed faster than non-cognates (words with different lexical representations between languages, i.e., “bed” in English and “cama” in Spanish). This evidence comes from studies in which the words were presented in the visual (e.g., Dijkstra et al., 1999; Costa et al., 2000; Hoshino and Kroll, 2008; Peeters et al., 2013) and the auditory modalities (Andras et al., under review; Woutersen et al., 1995; Bowers et al., 2000). Cognate facilitation has also been reported in spoken word production studies (Costa et al., 2005; see also Muscalu and Smiley, 2018 for typing). Thus, most models of bilingual language processing assume that both languages are co-activated and include predictions for the role of cognate words during word recognition (e.g., bilingual interactive activation BIA+ model; Dijkstra and Van Heuven, 2002) and word production (e.g., The revised hierarchical model – RHM; Kroll et al., 2010).

However, hypotheses regarding the processing of non-identical but similar cognates are not completely clear (Dijkstra et al., 2010). Cognate facilitation seems to be greater for identical cognates than non-identical cognates (Comesaña et al., 2015; Guasch et al., 2017) with larger cognate facilitation effects for words with greater orthographic similarity (OS; Dijkstra et al., 2010). Importantly, cognate words do not only differ in terms of OS between languages, but also in the degree of phonological overlap across languages. Recent models, such as the bilingual spelling in alphabetic systems (BAST) model (Tainturier, 2019), propose that the strength of co-activation is mediated by the degree of orthographic and phonological similarity (PS) between the two languages. However, the combined contributions of OS and PS have received little attention.

**Figure 1:**
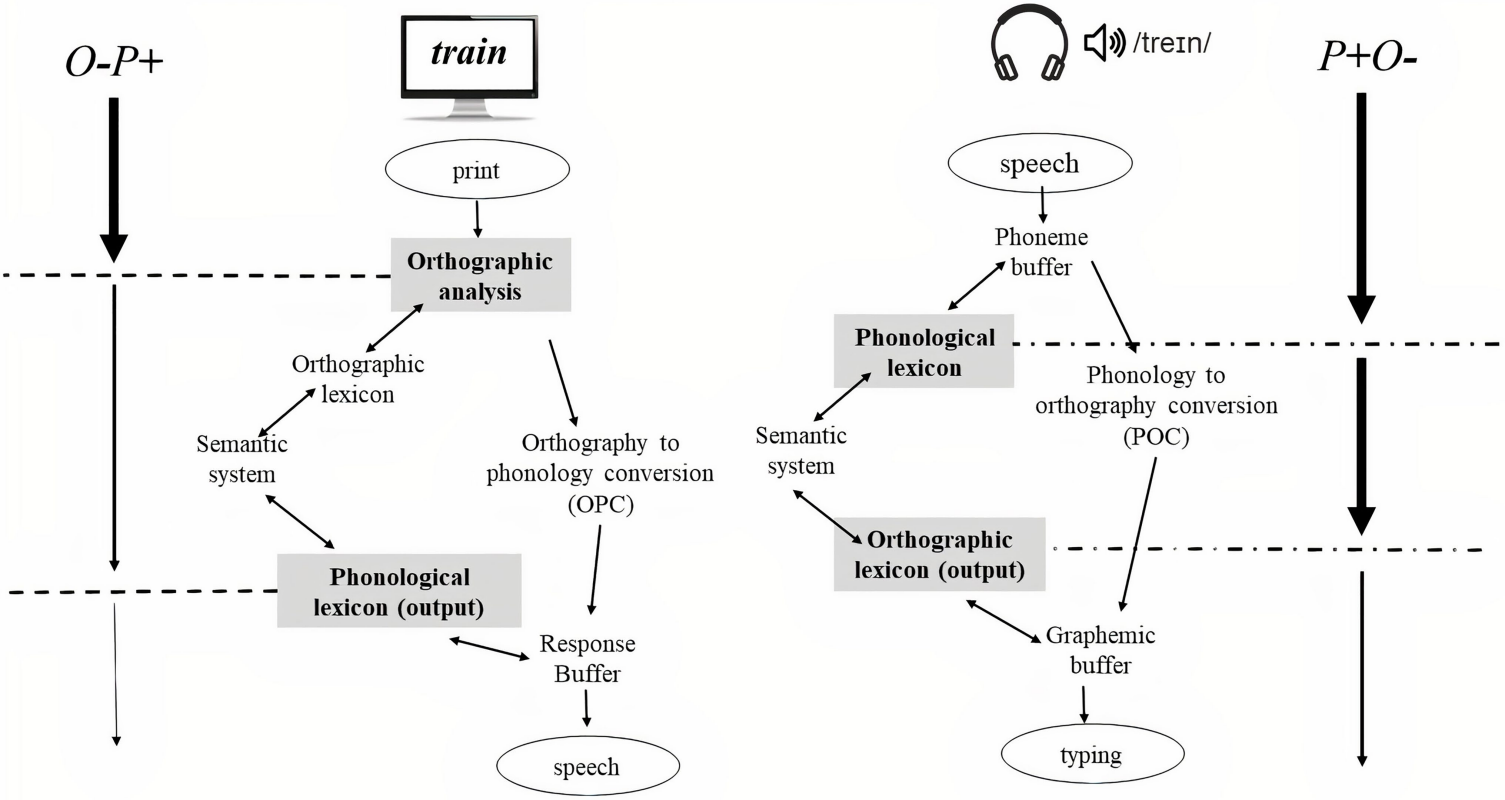
Reading vs. dictation to writing differences. In reading, the input is a string of letters, so the first analysis is orthographic. In the low OS condition, the representations of the two languages greatly differ, and therefore, they compete for selection. This orthographic analysis may act as a filter for cross-linguistic competition reducing the spread of activation so that non-target phonological information receives minimal activation (in the figure, the thickness of the left arrow is reduced as the processing progresses to represent this idea). On the contrary, in writing to dictation (current study), the input is auditory, so the first analysis is phonological. In this context, phonology has a direct impact on performance since there is not an orthographic filter to reduce the spread of activation to the non-target phonology (in the figure, the thickness of the right arrow is regular before and after the phonological filter). POC, phonology to orthography conversion system; OPC, orthography to phonology conversion system.

Most studies focusing on the interplay between OS and PS have been conducted using reading paradigms using strings of letters on the screen (Schwartz et al., 2007; Comesaña et al., 2012). The fact that the presented input is orthographic can undermine the possible role of phonology on language processing. According to cognitive models of reading (e.g., the dual-route model of reading; Coltheart et al., 2001), a visual stimulus may be decoded through the orthography to phonology conversion (OPC) system where a mapping between graphemes and phonemes occurs (letter-sound correspondence rules). Thus, during silent reading, phonology is activated, but its activation is delayed with respect to the first orthographic analysis. As such, in these kind of reading tasks, processing may be biased toward orthographic decoding. Conversely, writing production paradigms, and especially the writing to dictation task, can provide a useful tool to study the role of phonology and its interplay with orthography. In a writing to dictation task, the first input is phonological [phonology to orthography conversion (POC) system], due to words that are presented by auditory modality (e.g., the dual-route of spelling; Houghton and Zorzi, 2003) and therefore, orthographic activation occurs later than phonological activation (see Figure 1).

An effective approach to study the interplay of OS and PS could be the orthogonal manipulation of both variables. Comesaña et al. (2012) divided the cognate condition into four experimental conditions depending on the degree of orthographic and phonological similarity: O+P+ (*bomba*-BOMB), O+P− (*cometa*-COMET), O−P+ (*dança*-DANCE), and O−P− (*laço-LACE*), where the sign “+” indicates high overlap between languages, and the sign “-” indicates low overlap. Twenty-four Portuguese-English bilinguals performed a silent reading task including cognate and non-cognate words during a masked priming paradigm. Participants had to press the space bar to proceed to the next word (i.e., a self-paced reading task). Overall, performance (reaction times) was better for non-cognates than for cognates. Phonological effects were also present but they depended on the degree of orthographic similarity. Thus, cognates with high PS were read faster than cognates with low PS, but these differences were restricted to the high OS conditions (O+P+ vs. O+P−). For low OS cognates, the effect of phonology disappeared. In another study, Schwartz et al. (2007) asked English-Spanish bilinguals to read aloud cognates and non-cognates in both languages in two counterbalanced blocks. The orthogonal manipulation of orthographic and phonological similarity was also included as: O+P+ (*hospital*-HOSPITAL), O+P− (*genuino*-GENUINE), O−P+ (*noción*-NOTION), and O−P− (*músculo-*MUSCLE). Reading latencies were slower for cognates relative to non-cognates, suggesting an interference effect (from the onset of stimulus presentation to the onset of articulation). In addition, cognate words with high orthographic and phonological similarity (O+P+) were named faster than cognates with high orthographic similarity but low phonological overlap (O+P−). However, there was no difference between O−P+ and O−P−. That is, when the OS between languages was low, there was no PS effect (faster responses for high PS cognates than for low PS cognates). Therefore, the co-activation of phonology seems to be OS-dependent (orthographic autonomy hypothesis; Rapp and Caramazza, 1997). Only when the OS between languages was high was the phonology activated. Importantly, this pattern of results was observed both in the L2 (Spanish block) and L1 (English block). Hence, cross-language influences were evident during reading in the weaker L2 but also in the stronger L1.

The goal of the current study is to investigate the role of cognate status in bilingual writing production using a writing to dictation task in which a phonological analysis is mandatory. Specifically, we (1) compared performance (reaction time and accuracy) for cognate and non-cognate words in a typing paradigm and (2) examined the effect of orthographic and phonological co-activation in writing performance. To our knowledge, this study is the first to test the effect of orthographic and phonological activation across languages during a writing to dictation task. The critical materials included in this experiment consisted of cognate and non-cognate words (extracted from Schwartz et al., 2007). We included also the orthogonal manipulation of OS and PS: O+P+; O+P−; O−P+; and O−P−. Following previous studies investigating bilingual word recognition, we expected that the cognate facilitation effect (e.g., Costa et al., 2005; Hoshino and Kroll, 2008; Lemhöfer et al., 2008; Dijkstra et al., 2010) would be modulated by orthography, and more importantly also by the phonological overlap across languages. As in Schwartz et al. (2007), we expected that O+P+ would be typed faster than O+P− cognates, as evidence that phonological information is processed. However, in contrast to previous results, we also expected differences when the orthographic forms of cognates were different (O−P+ vs. O−P−), due to the differences between experimental tasks (see Figure 1). Different from reading studies in which the phonology only has an effect in high OS conditions, in our writing to dictation task, we predicted that the phonology would have an effect for high OS as well as for low OS conditions (significant differences between P+ and P−). In writing to dictation, the first input is phonological, so the phonological processing precedes orthographic processing, and therefore, the phonology would have a direct impact on performance. In this case, the phonological processing would be relatively independent of the orthographic overlap.

In addition to variations in the type of task, phonological and orthographic co-activation may also be dependent on the previous language experience of the bilingual participants. Previous studies have shown that the relationship between L1 and L2 is influenced by L2 competence and by the language learning background (Kroll et al., 2006; Dijkstra et al., 2010). Language experience is characterized by high variability on a range of factors related to language exposure and use (Green and Abutalebi, 2015; Anderson et al., 2018). The nature of the input received during learning has important consequences on language processing (Kroll et al., 2018; Fricke et al., 2019) and language outcomes (Place and Hoff, 2011; Byers-Heinlein, 2013). The quantity, and, even more important, the quality of the input are strong predictors of the language development in bilinguals (Gathercole and Thomas, 2009). In this context, it is fundamental to consider differences between naturalistic and classroom settings (Rothman and Guijarro-Fuentes, 2010). It is well known that L2 learners in a classroom setting receive considerably less oral input than in a naturalistic setting (and of course than native speakers). Qualitative differences in input during learning might serve to explain some asymmetries between L2-learners in classroom and naturalistic environments. The learning background might be especially relevant when examining bilingual writing because writing competence might differ depending on whether L1 or L2 was formally acquired at school, or whether it was learned and used at home where verbal/auditory input exceeds visual/written exposure. These differences could have an important impact on the interplay of orthographic and phonological processing.

In order to address this critical question, we included two groups of English-Spanish bilinguals with different language learning backgrounds: native English speakers who were Spanish learners [late bilinguals (LBs) with formal education in Spanish] and Spanish heritage speakers (HSs) who had acquired English and Spanish at an early age in the household but did not receive a formal education in Spanish. Both groups of participants were immersed in an English dominant context and immersed in English education. The selection of these two groups provides the opportunity for examining the effects of phonological and orthographic co-activation in cognate writing production by English-Spanish bilinguals, who have different background experiences in one of their languages, experience with academic literacy and formal instruction in Spanish and English (LBs) vs. experience with academic literacy and formal instruction just in English (HSs; Carrasco-Ortiz et al., 2019). L1 acquisition is normally characterized by being homogeneous, systematic, and complete. However, the L1 acquisition in the HSs could be unstable and incomplete (Montrul, 2008; Polinsky, 2011). As HSs learn their minority language (L1) at home, and at the same time, they are immersed in a majority language (L2) context (Benmamoun et al., 2013), they receive mainly oral/phonological input during L1-learning (in a naturalistic environment). In contrast, L2 learners are exposed to formal education of reading and writing, but also to oral inputs in an instructed context (e.g., Hyltenstam and Abrahamsson, 2003; Paradis, 2004). Given the higher exposure to oral/phonological input in HSs in comparison with L2 learners, HSs are thought to have a phonological advantage (Chang et al., 2011; Gor, 2014). In addition, studies have also pointed out difficulties in orthographic knowledge in HSs (Elola and Mikulski, 2016) especially during writing tasks (Montrul, 2013). These described differences across bilingual speakers made it possible to expect stronger phonological effects in the HSs than in LBs (faster responses for cognates with high PS than for cognates with low PS), especially during English writing, in which the influence of Spanish phonology is expected. In addition, stronger orthographic effects were expected for LBs relative to HSs, especially during English writing due to their greater familiarity with Spanish orthography. Note that “stronger phonological effects” mean higher differences between P+ and P− conditions. On the contrary, “stronger orthographic effects” mean higher differences between O+ and O− conditions.

## Materials and Methods

### Participants

Forty-eight bilingual students from the University of Florida (United States) participated in the study in exchange for partial course credit. One participant was excluded because he reported central auditory processing disorder. The remaining 47 participants reported normal hearing and normal vision, and they did not report any language or neurological deficits. All participants were able to type using their 10 fingers. They were classified into two experimental groups: 23 LBs and 24 HSs. Both groups were immersed in an English dominant context and they had been educated in the United States.

As data analysis was implemented as mixed-effect regression analysis, we checked if our observations were enough for this type of analysis. Brysbaert and Stevens (2018) recommend “at least 1.600-word observations per condition (e.g., 40 participants, 40 stimuli).” In the current study, observations from 47 participants (23 LBs and 24 HSs) and from 208 words (104 cognates vs. 104 non-cognates) were included. This resulted in 2392 observations for the LBs, and 2,496 observations for the HSs in each condition. However, some of these observations were excluded from analysis due to the data trimming performed to eliminate outliers (see “Results”). Despite this, we had enough observations, with 2,104 observations remaining in the LBs (and 2,170 for non-cognates), and 2,242 observations in the HSs (and 2,238 for non-cognates). This estimation is similar to the ones reported previous studies (Schwartz et al., 2007; Comesaña et al., 2012).

To determine their language dominance and background experiences (experience with academic literacy and formal instruction), all participants completed the language experience and proficiency questionnaire (LEAP-Q; Marian et al., 2007) for both languages, Spanish and English. Table 1 summarizes the language use and exposure data and the proficiency level of the participants.

**Table 1:** Mean scores (with standard deviation in parenthesis) for English and Spanish language experience in the LBs and HSs.

*Language version*		English (L1/majority language)			Spanish (L2/minority language)		
		LBs (*N* =23)	HSs (*N* =24)		LBs (*N* =23)	HSs (*N* =24)	
*LEAP-Q items*				*p*			*p*
AoA		0.74 (0.91)	2.71 (1.87)	^**^	10.69 (3.61)	0.92 (1.32)	^**^
Years of exposure	Country	19.91 (1.16)	18.71 (2.62)	^*^	0.13 (0.62)	6.08 (8.35)	^**^
	Family	19.65 (1.99)	16.54 (6.57)	^*^	0.87 (2.41)	19.45 (1.59)	^**^
	School	17.95 (2.94)	16.50 (2.87)		4.56 (5.01)	3.33 (5.94)	
Self-assessed capacity (from 1 to 10)	to speak	9.69 (0.55)	9.54 (0.77)		6.30 (1.22)	7.08 (1.21)	^*^
	to understand	9.60 (0.78)	9.71 (0.55)		7.35 (1.26)	8.25 (1.22)	^*^
	to read	9.65 (0.57)	9.66 (0.63)		7.26 (1.54)	7.08 (1.47)	
Reading contribution to learning		8.89 (1.42)	8.71 (1.49)		7.35 (2.27)	5.87 (2.69)	^*^
*Spanish Writing and Spelling tests*							
PROESC					22.43 (1.87)	20.83 (2.91)	^*^
TECLE					35.74 (7.06)	31.96 (5.20)	^*^

AoA, age of acquisition; LBs, late bilinguals; and HSs, Heritage speakers

* *p<* 0.05;

** *p<* 0.01.

The LEAP-Q data show that the LBs were exposed to English earlier than the HSs [age of first exposure (AoA), *t* (45)=−4.541, *p*<0.001] because they were born into an English-speaking country/family and context. In addition, LBs spent more years in an English-speaking country, *t* (45)=2.016, *p*=0.049, and LBs spent more years living in a familiar English environment, *t* (45)=2.177, *p*=0.035 than HSs. Importantly, the difference in years of exposure to school context in English was not significant, *t* (45)=1.716, *p*=0.093. Importantly, the difference in the self-assessed English skills was not significant (speaking, understanding, and reading; all *ps*>0.05). In order to explore the instructed context of English learning, we analyzed the specific item *reading contribution to learning* (see the question 4 in the LEAP-Q questionnaire: “Please mark how much the following factors contributed to you English/Spanish learning”). The participants rated this item on a scale of 1 to 10. Low scores indicate that reading has contributed little to their learning. This score reflects the degree of formal language education in one language, which is thought to be an important requirement for correct learning of orthography (Iniesta et al., 2021). The differences between groups were not significant; *t* (45)=−0.030, *p*=0.976.

Conversely, HSs were exposed earlier to Spanish (AoA) than LBs [*t* (45)=8.467, *p*<0.001] because they were born into a Spanish-speaking family. In addition, HSs lived longer than LBs in a Spanish-speaking country [*t* (45)=−3.408, *p*=0.001] and familiar Spanish environment [*t* (45)=−31.287, *p*<0.001]. Overall, HSs presented greater exposure to Spanish. However, the difference with respect to years of exposure to school context in Spanish was not significant [*t* (45)=0.767, *p*=0.447]. The difference in the self-assessed Spanish skills was significant for speaking [*t* (45)=−2.193, *p*=0.034] and understanding [*t* (45)=−2.484, *p*=0.017]. The HSs scored higher on these scales, as expected. However, in the skill more related to formal use of language, reading [*t* (45)=0.404, *p*=0.688], there were no differences between groups. As for English, we explored the *reading contribution to learning* for Spanish revealing that HSs had a significantly lower score [*t* (45)=2.024, *p*=0.048].

In addition to the self-rated questionnaire, participants also completed a formal standardized test in Spanish for writing and spelling (PROESC – Batería de Evaluación de Los Procesos de Escritura; Cuetos et al., 2002). As part of PROESC, participants completed the ruled-orthography subtest consisting of a pen and paper writing to dictation task of 25 words that included a Spanish spelling rule (Chacón, 1997). For example, in Spanish, all verbs that end in -aba (i.e., cantaba) are spelled with “b” instead of “v.” In addition, all words that end in -aje (chantaje) are spelled with “j” instead of “g.” In addition, participants completed a silent efficiency reading test (TECLE – Test de Eficiencia Lectora; Marín and Carrillo, 1999) including an orthographic decision subtest in which there were sentences with one word missing. Participants had to select the correct word, among 4 options that included semantic, spelling, and phonological distractors, which included subtle letter changes. In 3min, the participant had to solve the maximum number of sentences as possible among a total of 64 sentences. A good knowledge of spelling is necessary to select the correct option. The results showed better accuracy in word writing in PROESC for the LBs (mean=22.43; *SD*=1.87) than the HSs (mean=20.83; *SD*=2.91) out of 25 words in total, *t* (45)=2.228, *p*=0.031. Additionally, the LBs were more accurate in the TECLE than the HSs (LBs: mean=35.74; *SD*=7.06; HSs: mean=31.96; *SD*=5.20); *t* (45)=2.095, *p*=0.042.

These results confirmed that, despite the higher speaking and understanding abilities that HSs reported for Spanish in the self-reported questionnaire, no differences in reading skills were evidenced (the fact that in HSs the superiority in speaking and understanding was not extended to reading could indicate the lower skills with the formal aspect of Spanish). Additionally, the LBs had higher orthographic knowledge of Spanish than the HSs in formal standardized test. This provides support to the assumption that HSs might be biased toward phonology and that they might have more difficulties with the more formal aspects of Spanish (including orthographic rules), due to their informal learning background.

### Materials

A total of 208 words in English and their Spanish translations were selected (extracted from Schwartz et al., 2007). English and Spanish items were presented in two independent blocks. Each language block (Spanish or English) was comprised of 104 cognates and 104 non-cognates. Schwartz et al. (2007) classified them according to the OS score (Van Orden et al., 1988; Yates et al., 2003). If the OS was higher than 0.3, this word was classified as cognate. The conditions were matched in logarithmic lexical frequency and the number of letters (Guasch et al., 2013), age of acquisition (AoA; Kuperman et al., 2012; Alonso et al., 2015), concreteness (Duchon et al., 2013; Brysbaert et al., 2014), and orthographic and phonological neighbors (Marian et al., 2012). English-Spanish cognates and non-cognates were presented aurally. The experimental material was read by a female Puerto Rican Spanish-English bilingual. The material was recorded using a Shure SM57 microphone on a Marantz Solid State Recorder PMD670 (Valdés Kroff et al., 2019). The recorded items were then isolated using a script implemented in PRAAT software (version 5.3.16; Boersma and Weenink, 2012) employing *TextGrids* for segmentation and labeling. In addition, the script added 50ms of silence at the beginning and 500ms at the end of each word by default, and it resampled the words so that they were at 44.1kHz in monoaural. It also rescaled and equated the loudness of the files. Table 2 shows descriptive statistics for the experimental material.

**Table 2:** Characteristics of the experimental stimuli (mean scores with standard deviations in parenthesis).

	Within-language variables						
	Frequency	Letters	Concreteness	AoA	PN	ON	Audio
English block							
Non-cognates	1.41 (0.47)	6.09 (1.26)	3.92 (1.10)	6.53 (2.01)	6.93 (6.99)	4.01 (5.21)	647 (144)
Cognates	1.42 (0.48)	5.94 (1.41)	3.69 (1.01)	6.95 (2.25)	5.48 (7.59)	3.50 (4.11)	667 (124)
*Statistics*	*t* (206)=0.188, *p* =0.851	*t* (206)=−0.837, *p* =0.404	*t* (206)=−1.531, *p* =0.127	*t* (206)=1.43, *p* =0.154	*t* (206)=−1.42, *p* =0.157	*t* (206)=−0.779, *p* =0.437	*t* (206)=1.08, *p* =0.203
***O+P+***	1.50 (0.46)	6.11 (1.51)	3.91 (0.91)	6.98 (1.47)	5.75 (7.68)	3.35 (3.95)	700 (150)
***O+P–***	1.35 (0.48)	5.87 (1.49)	3.53 (0.97)	7.02 (2.67)	5.13 (8.62)	4.03 (4.45)	632 (89)
***O – P+***	1.43 (0.34)	5.89 (0.87)	3.80 (1.24)	7.24 (2.04)	4.53 (5.25)	3.36 (2.26)	698 (112)
***O – P–***	1.41 (0.61)	5.88 (1.58)	3.56 (0.93)	6.62 (2.61)	6.30 (6.93)	3.84 (4.51)	652 (127)
*Statistics*	*F* (3, 104)=0.433, *p* =0.729	*F* (3, 104)=0.171, *p* =0.916	*F* (3, 104)=0.910, *p* =0.439	*F* (3, 104)=0.299, *p* =0.826	*F* (3, 104)=0.232, *p* =0.874	*F* (3, 104)=0.722, *p* =0.541	*F* (3, 104)=1.897, *p* =0.204
Spanish block							
Non-cognates	1.37 (0.47)	6.65 (1.72)	4.98 (1.42)	5.28 (2.35)	3.15 (4.45)	2.89 (3.42)	663 (142)
Cognates	1.37 (0.54)	6.27 (1.53)	4.74 (0.98)	5.85 (3.06)	3.10 (3.88)	2.66 (3.26)	673 (128)
*Statistics*	*t* (206)=−1.211, *p* =0.227	*t* (206)=−1.648, *p* =0.101	*t* (206)=−1.21, *p* =0.232	*t* (206)=0.702, *p* =0.484	*t* (206)=−0.074, *p* =0.941	*t* (206)=−0.492, *p* =0.623	*t* (206)=0.516, *p* =0.607
***O+P+***	1.48 (0.42)	6.35 (1.59)	4.87 (0.91)	5.90 (2.16)	2.96 (3.13)	2.71 (2.59)	698 (146)
***O+P–***	1.39 (0.64)	6.16 (1.55)	4.73 (1.14)	5.40 (3.70)	2.53 (3.23)	2.53 (2.78)	657 (119)
***O – P+***	1.27 (0.55)	6.21 (1.03)	4.73 (1.11)	6.43 (2.55)	2.94 (3.09)	1.52 (1.64)	651 (111)
***O – P–***	1.42 (0.48)	6.58 (2.13)	4.62 (0.89)	5.56 (2.86)	3.11 (3.88)	2.61 (3.23)	680 (132)
*Statistics*	*F* (3, 104)=0.573, *p* =0.634	*F* (3, 104)=0.335, *p* =0.800	*F* (3, 104)=0.227, *p* =0.877	*F* (3, 104)=0.550, *p* =0.649	*F* (3, 104)=0.757, *p* =0.521	*F* (3, 104)=1.496, *p* =0.220	*F* (3, 104)=0.747, *p* =0.526

AoA, age of acquisition; PN, phonological neighbors; ON, orthographic neighbors; Audio, audio duration; +, high similarity; and −, low similarity

As in Schwartz et al. (2007) the cognates condition also included the orthogonal manipulation of OS and PS including high (+) and low (−) similarity: O+P+ (*n*=28); O+P− (*n*=31); O−P+ (*n*=19); and O−P− (*n*=26). If the OS was greater than 0.70, the cognate word was classified as high similarity condition. Otherwise, it was classified as low similarity. The PS was calculated subjectively using the following procedure. Pairs of cognate words were auditorily presented to the participants (English monolinguals). The pairs were recorded and spoken by two fluent bilinguals with each member of the pair spoken by a different bilingual. Participants (*n*=29) rated the phonological similarity of cognate pairs on a Likert scale from 1 (no similarity) to 7 (very similar). If the PS was greater than 4, the cognate word was classified as high similarity. Otherwise, it was classified as low similarity (we report norming that were conducted and reported by Schwartz et al., 2007). Table 3 shows the OS and PS for each condition. Also see Table 2 for the information about frequency, number of letters, age of acquisition, concreteness and neighbors relative to these four experimental conditions.

**Table 3:** Orthographic and phonological similarity across experimental conditions (mean scores with standard deviations in parenthesis).

		Cross-linguistic variables	
		OS	PS
Non-cognates		0.14 (0.09)	–
Cognates		0.74 (0.23)	–
*Statistics*		*t* (206)=24.21, *p* =0.000	
(1) O+P+		0.92 (0.12)	5.31 (0.91)
(2) O+P−		0.88 (0.13)	2.84 (0.67)
(3) O−P+		0.50 (0.13)	5.02 (0.71)
(4) O−P−		0.54 (0.17)	2.84 (0.73)
*Statistics*		*F* (3, 104)=60.45, *p* =0.000	*F* (3, 104)=81.34, *p* =0.000
*Post-hoc comparisons*	1 vs. 2	*t* (57)=0.931, *p* =0.356	*t* (57)=11.87, *p* =0.000
	1 vs. 3	*t* (45)=11.11, *p* =0.000	*t* (45)=1.209, *p* =0.233
	1 vs. 4	*t* (52)=9.27, *p* =0.000	*t* (52)=10.91, *p* =0.000
	2 vs. 3	*t* (48)=9.84, *p* =0.000	*t* (48)=−10.82, *p* =0.000
	2 vs. 4	*t* (55)=8.406, *p* =0.000	*t* (55)=−0.005, *p* =0.996
	3 vs. 4	*t* (43)=−0.963, *p* =0.341	*t* (43)=9.96, *p* =0.000

OS, orthographic similarity; PS, phonological similarity. We report norming that were conducted and reported by Schwartz et al. (2007). Data from non-cognates words were not available in the original research.

### Procedure

**Figure 2:**
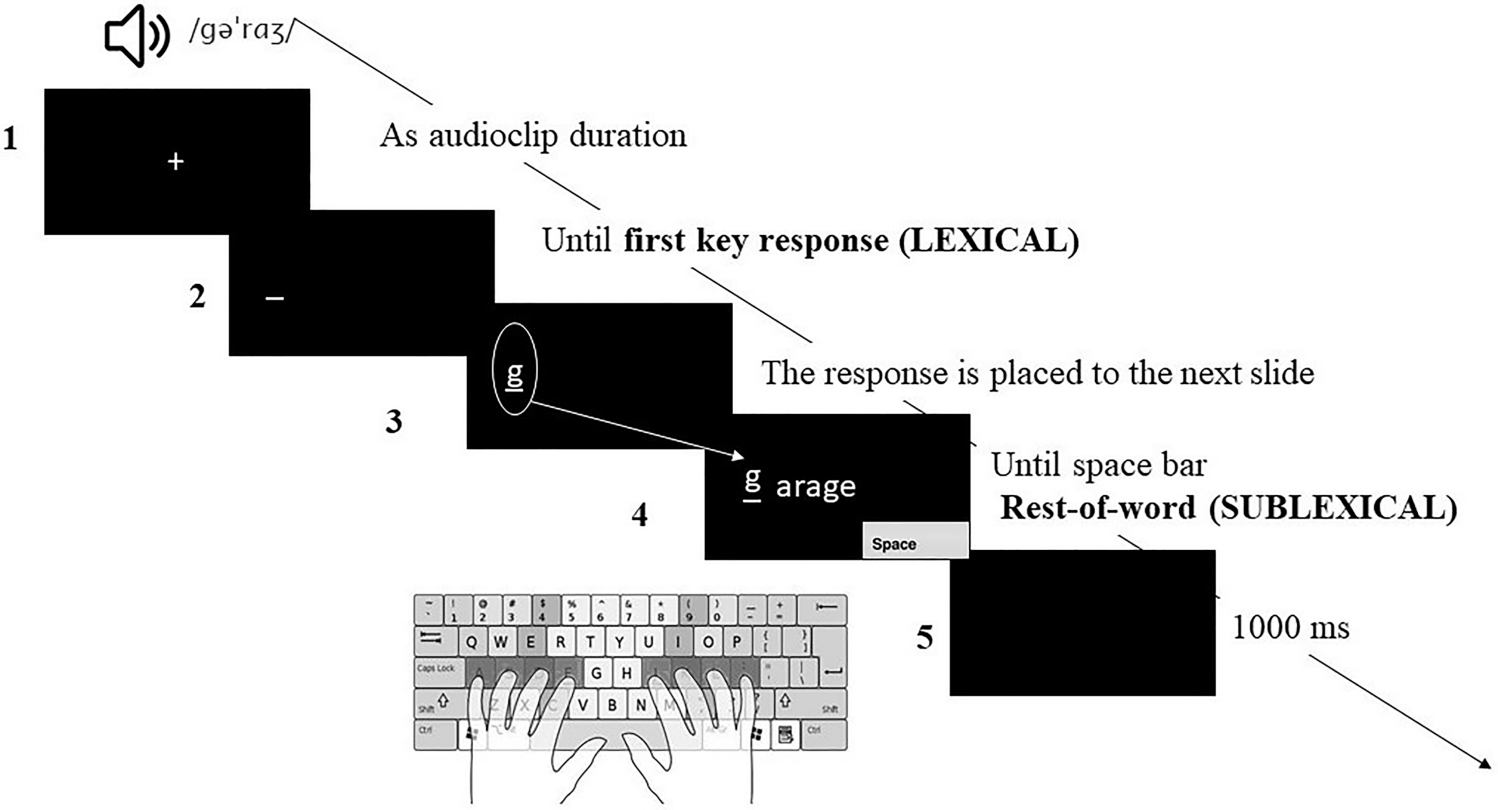
An example of an experimental trial. Participants typed the whole word. The first keypress (first key response-lexical) and the latency of the rest of the word (rest of word response sublexical) were recorded. /gəˈrɑʒ/ represents the phonetic transcription of garage following the Carnegie Mellon University Pronouncing Dictionary. The numbers 1 to 5 have been associated with the description of the procedure in the main text. Point 3 (the response is placed to the next slide) refers to the programming aspect. We used the (response. RESP) E-prime attribute to automatically register the participant’s response from the previous slide (lexical latency) and to continue recording the participant’s response until the end (sublexical latency), but participants were unaware of this feature of the display and perceived their typing as continuous.

After signing the consent form, participants in both groups performed the writing to dictation task in two independent blocks (Spanish and English). The order of presentation was counterbalanced between participants. The items were randomized (the four conditions of cognates and the condition of non-cognates). Each block began with eight practice trials, followed by the experimental block, with 208 trials in each language. We included a break in the middle of each block, with a duration adaptable to the needs of the participant. The writing to dictation task was conducted on a computer using E-prime version 3.0 (Psychology Software Tools, Pittsburgh, PA). Participants wore headphones to listen to the stimuli and used a standard QWERTY keyboard to type words. Each trial (see Figure 2) started with a fixation point (1) which remained on the screen until the auditory stimulus was presented. As soon as the audio terminated, a position bar (2) appeared on the screen indicating that the participants could start to write. Typing was not enabled until the appearance of this position bar. Participants were instructed to type as quickly and accurately as possible. The responses appeared on the screen at the same time as participants were writing.

Importantly, language co-activation in cognate words could be evidenced as facilitation or interference depending on whether co-activation occurs at a lexical or sublexical level (Muscalu and Smiley, 2018; Iniesta et al., 2021), or depending on whether co-activation occurs in a more initial and central process (lexical retrieval), or in a more posterior or peripheral process (Purcell et al., 2011). For this reason, the reaction time (RT) and accuracy (ACC) of the typing response were collected in two different temporal moments associated with lexical and sublexical processing (see Muscalu and Smiley, 2018 and Iniesta et al., 2021 for a similar procedure): from the offset of the stimulus to the first keypress (first key performance) (3) and from the first keypress to the press of the space bar key (rest of the word performance) (4). These two measures have been associated with lexical and sublexical processing, respectively, and therefore allowed us to pinpoint the time course and level of linguistic analysis at which our effects occurred. Considering that the experiment was carried out with an English keyboard, the participants received explicit instructions not to write the diacritical marks during the Spanish block. In addition, one word included a “ñ” grapheme. The participants were instructed to press the key adjacent to the “l,” which would be the natural position of the ñ on a Spanish keyboard. Between trials, there was a black screen for 1000ms (5).

Between the English and Spanish blocks of the writing to dictation task, participants completed the LEAP-Q questionnaire (Marian et al., 2007) for both languages (Spanish and English), and the two Spanish assessment tests (PROESC and TECLE, see the participants section, for more information). Overall, the experimental session lasted approximately 60min. The study was conducted in accordance with the ethical standards approved by the University of Florida Institutional Review Board (IRB): protocol #2019–02427.

## Results

For the writing to dictation task, the ACC and the RTs for correct responses were calculated for each participant and condition for the first keystroke and the rest of the word separately. Response times above or below 2.5 SD from each participant’s mean were eliminated from the analysis [first key performance: 3.31% (English)/4.39% (Spanish) of the items of the LBs and 3.92% (English)/4.49% (Spanish) from the HSs; rest of the word performance: 4.15% (English)/4.87% (Spanish) of the items of the LBs and 4.88% (English)/5.58% (Spanish) from the HSs]. Accuracy was determined based on a strict criterion for correct (1) vs. incorrect (0) scores. Clear typographical errors were also considered as correct (e.g., helic***q***opter. In this case, the key “q” is not necessary and it is not surrounding any target key). In the same way, errors derived from accentuation in Spanish were also considered correct. Although the instructions explicitly indicated not to type the accent marks, some participants made mistakes trying to type them, and we also considered these words as correctly typed (e.g., m***^***aquina, the Spanish word for machine). Note that there were only eight observations in this special situation.

Following previous studies, two independent analyses were conducted to explore (Schwartz et al., 2007; Comesaña et al., 2012): (1) the *overall effect of language and cognate status* in the performance of both groups of participants and (2) *the impact of OS and PS in cognates*.

A mixed-model analysis was performed using the R lme4 package (R Core Team, 2017; Bates et al., 2015) and including the function with a “Kenward-Roger” modification for *F*-tests (Halekoh and Højsgaard, 2014) in order to include the random effects in the analysis (Luke, 2017). The model for the first analysis (overall effect of language and cognate status) was conducted with Group (LBs vs. HSs), Language (English vs. Spanish), and Condition (Cognates vs. non-cognates) as fixed factors and Participants and Items as random effects for *first key* and *rest of the word* performances. For the second analysis (the impact of OS and PS), the model included Group (LBs vs. HSs), Language (English vs. Spanish), OS (+ vs. −), and PS (+ vs. −) as fixed factors and Participants and Items as random effects both for *first key* and *rest of the word* performances. Participants and Items were included as random intercepts, random slopes were not included because a simplification of the maximal following the convergence of models (Barr et al., 2013). When a two-way interaction was found, a post-hoc *t*-test using Tukey’s multiple comparison correction was implemented using the R function *lsmeans*. When a three-way interaction (or above) was significant, a new model exploring this specific interaction was performed, also including participants and items as random effects. Finally, *p*-values were reported by the *anova* function of the LmerTestR-package. Full models’ summary is available from the OSF repository: https://osf.io/bkhvj/?view_only=325b38c094ff41749f2db2a9ef608286.

### The Overall Effect of Language and Cognate Status

Table 4 summarizes the results (RTs and ACC) obtained in the writing to dictation task as a function of Group (LBs vs. HSs), Language (English vs. Spanish), and Condition (cognates vs. non-cognates).

**Table 4:** Mean scores (with standard errors in parenthesis) in the writing to dictation for the overall effect of language and cognate status in each participant group (analysis 1).

		First key				Rest of word			
		English		Spanish		English		Spanish	
		LBs	HSs	LBs	HSs	LBs	HSs	LBs	HSs
RTs	Cognates	643 (29.8)	713 (29.3)	764 (29.8)	744 (29.3)	1182 (58.1)	1325 (57.3)	1318 (58.0)	1550 (57.3)
	Non-cognates	562 (31.3)	635 (30.9)	753 (31.4)	768 (31.0)	1085 (63.2)	1254 (62.4)	1549 (63.3)	1852 (62.6)
ACC	Cognates	0.944 (0.009)	0.946 (0.009)	0.943 (0.009)	0.956 (0.008)	0.832 (0.019)	0.817 (0.018)	0.868 (0.019)	0.841 (0.018)
	Non-cognates	0.966 (0.010)	0.971 (0.010)	0.924 (0.010)	0.944	0.898 (0.021)	0.884 (0.021)	0.829 (0.022)	0.812 (0.022)

#### First Key Performance

##### Latency

For *first key* latencies (RTs), the main effect of Language was significant, *F* (1, 478.9)=76.38, *p*<0.001. Responding in English (mean=638ms) was faster than responding in Spanish (mean=757ms). The main effect of Condition was also significant, *F* (1, 349.4)=3.95, *p*=0.047. Cognates were responded to slower (mean=716ms) than non-cognates (mean=679ms).

The interaction between Group and Language was also significant [*F* (1, 17185.1)=51.54, *p*<0.001]. For both groups, the differences between Spanish and English were significant [LBs: *t* (17182.3)=−10.67, *SE*=14.6, *p*<0.001; HSs: *t* (17186.4)=−5.66, *SE*=14.5, *p*<0.001], but the magnitude of the differences was greater in the LBs (Spanish: 759–English: 602=157ms) than in the HSs (Spanish 756 – English: 674=82ms). The interaction between Language and Condition was also significant [*F* (1, 478.8)=9.93, *p*=0.002] with cognates being slower (mean=678ms) than the non-cognates [mean=598ms; *t* (477.2)=3.48, SE=23.0, *p*<0.001] in the English block. However, these differences were not significant in the Spanish block [mean of cognates=754ms; mean of non-cognates=760ms; *t* (475.2)=−0.26, SE=23.1, *p*=0.791]. No other main effects or interactions were significant (all *ps*>0.05).

##### Accuracy

For *first key* accuracy, there was a main effect of Group, *F* (1, 46.3)=4.25, *p*=0.045, with higher accuracy for HSs (mean=0.954) than for LBs (mean=0.944). The main effect of Language was also significant, *F* (1, 492.7)=4.22, *p*=0.040, such that accuracy in English (mean=0.957) was higher than in Spanish (mean=0.942).

A Group × Language interaction was also significant, *F* (1, 18076.1)=5.35, *p*=0.021. For LBs, the difference between English (mean=0.955) and Spanish (mean=0.945) was significant [*t* (18072.3)=2.74, *SE*=0.008, *p*=0.006], whereas for HSs, it was not [English mean=0.959; Spanish mean=0.950; *t* (18072.8)=1.09, *SE*=0.007, *p*=0.272]. The Language × Condition interaction was also significant, *F* (1, 492.7)=6.79, *p*=0.009, showing that for the English block, cognates (mean=0.945) were less accurate than non-cognates (mean=0.969), *t* (491.1)=−1.989, *SE*=0.012, *p*=0.046. In contrast, for the Spanish block, the difference between cognates and non-cognates was not significant [cognates mean=0.949, non-cognates mean=0.934; *t* (490.8)=1.19, SE=0.013, *p*=0.232]. No other main effects or interactions were significant (all *ps*>0.05).

#### Rest of the Word Performance

##### Latency

Regarding the RTs of the *rest of the word*, there was a main effect of Group, *F* (1, 46.9)=10.79, *p*=0.002. LBs (mean=1284ms) showed faster responses than the HSs (mean=1495ms). There was also a main effect of Language, *F* (1, 428.8)=124.66, *p*<0.001. The responses in English (mean=1211ms) were faster than Spanish (mean=1567ms). Similarly, the main effect of Condition was significant, *F* (1, 354.2)=4.107, *p*=0.043. Cognates (1344ms) were typed faster than non-cognates (1435ms).

The interaction between Group and Language, *F* (1, 15244.1)=38.35, *p*<0.001, was also significant. For both groups, the differences between Spanish and English were significant [LB: *t* (15238.1)=−9.05, *SE*=33.1, *p*<0.001; HS: *t* (15245.3)=−12.44, *SE*=33.1, *p*<0.001]. However, the magnitude of the difference was greater for the HSs (Spanish 1701 – English: 1289=412ms) than for the LBs (Spanish: 1434 – English: 1134=300ms). The interaction between Group × Condition was also significant, *F* (1,15242.3)=7.17, *p*=0.007. Thus, for LBs, there were no differences between cognates (mean=1250ms) and non-cognates [mean=1357ms; *t* (15241.6)=−1.46, *SE*=46.0, *p*=0.143], whereas these differences were significant in the HSs [mean of cognates=1437ms; mean of non-cognates=1553ms; *t* (15239.4)=−2.51, *SE*=46, *p*=0.012]. The interaction between Language and Condition was also significant, *F* (1, 428.6)=30.37, *p*<0.001, such that in the English block, there were no differences between cognates (mean=1254ms) and non-cognates [mean=1169ms; *t* (427.3)=1.52, *SE* =55.2, *p*=0.128], whereas in the Spanish block, cognates (mean=1434ms) were faster than non-cognates [mean=1701ms; *t* (428.4)=−4.83, *SE*=55.3, *p*<0.001]. The three-way interaction was not significant [Group × Language × Condition, *F* (1, 15242.5)=1.61, *p*=0.204].

##### Accuracy

For *rest of the word* accuracy, no main effects were significant; Group, *F* (1, 46.5)=2.25, *p*=0.139; Language, *F* (1, 443.9)=1.90, *p*=0.168; and Condition, *F* (1, 363.1)=0.63, *p*=0.427.

However, the Language × Condition interaction was significant, *F* (1, 443.9)=11.53, *p*<0.001. In the English block, cognates (mean=0.824) were less accurate than non-cognates (mean=0.891), *t* (442.6)=−2.618, *SE*=0.025, *p*=0.008, whereas the differences in the Spanish block were not significant [cognates mean=0.854, non-cognates mean=0.821; *t* (443.7)=1.315, *SE*=0.025, *p*=0.188]. No other interactions were significant (all *ps*>0.05).

#### Summary of the Language and Cognate Status Analysis

The *first key* responses were slower for Spanish (L2) than for English (L1), although this effect was modulated by subtle differences in language experience (e.g., LBs were slower in Spanish than in English to a greater extent than the HSs. In addition, LBs were more accurate in English than in Spanish, but these language differences in accuracy were not present in HSs). In addition, both groups showed similar patterns of cognate effects, with cognate interference being evident in English (L1), but absent in Spanish (L2), in latency, and accuracy. For the *rest of word*, response times differed for language, group, and condition: Responses were slower for Spanish (L2) than for English (L1), although this effect was modulated by the differences in language experience (e.g., HSs were slower in Spanish than in English to a greater extent than the LBs). Writing cognate words were faster than writing non-cognate words, but this facilitatory effect showed some nuanced relations with language (only present in Spanish when looking at response times). Importantly, the group-by-condition interaction indicated that the facilitatory effect was only present for the HSs. However, in English (L1), writing cognates were less accurate than writing non-cognate words in both groups, revealing a similar cognate interference effect to that found for the first key.

### The Impact of Orthographic (OS) and Phonological Similarity (PS) in Cognates

**Figure 3:**
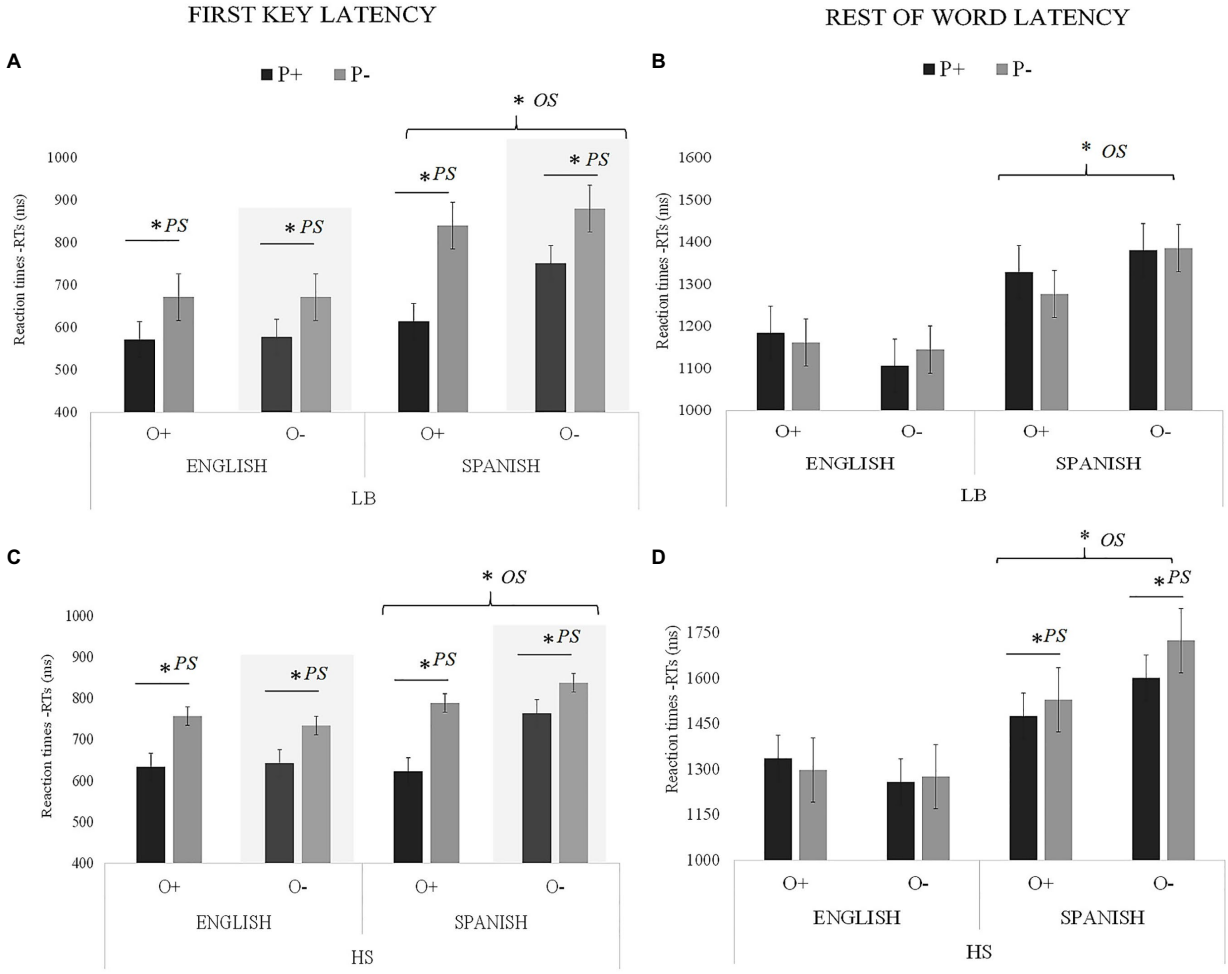
Visual representation of OS and PS latency results for the cognate condition (milliseconds): **(A)** LBs first key; **(B)** LBs rest of the word; **(C)** HSs first key; and **(D)** HSs rest of the word. Asterisks next to PS indicate significant effects of phonology, and asterisks next to OS indicate significant effects of orthography.

**Figure 4:**
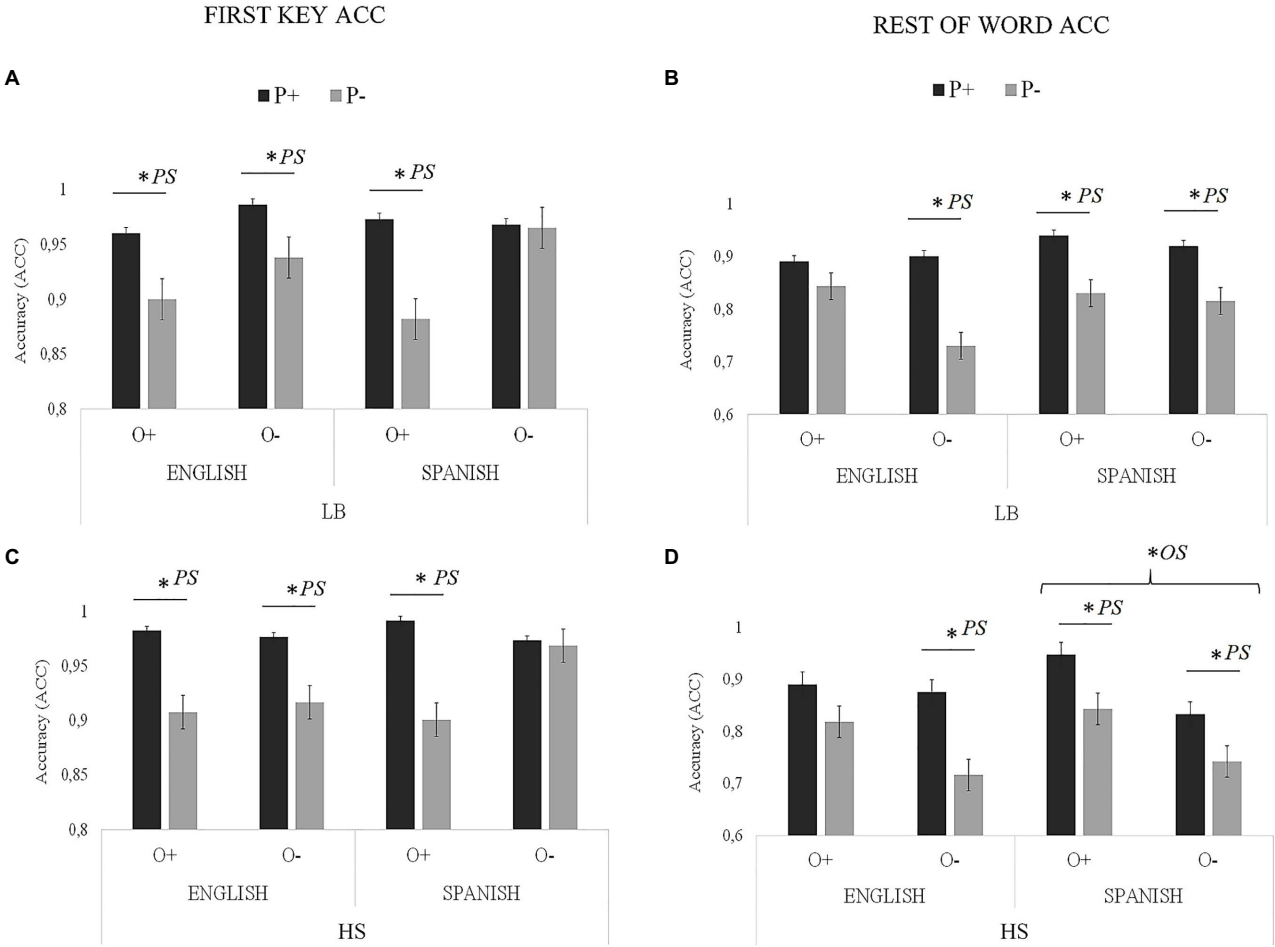
Visual representation of OS and PS accuracy results for the cognate condition (proportion of correct responses): **(A)** LBs first key; **(B)** LBs rest of the word; **(C)** HSs first key; and **(D)** HSs rest of the word. Asterisks next to PS indicate significant effects of phonology, and asterisks next to OS indicate significant effects of orthography.

Figure 3 (for latency) and Figure 4 (for accuracy) summarize the results obtained in the writing to dictation task in relation to a new analysis including four factors: Group (LBs vs. HSs), Language (English vs. Spanish), OS (High vs. Low), and PS (High vs. Low) within the cognate condition. In addition, a summary of statistics has been included in Table 5 (main effects and interactions). In the following subsections, we further analyze the significant effects reported in Table 5.

**Table 5:** Summary of results (main effects and interactions) of the OS and PS in the cognate words condition (analysis 2).

	First key		Rest of the word	
*Effects*	Latency	ACC	Latency	ACC
*Group*	*F*(1, 47)=0.42, *p* =0.515	*F*(1, 48)=1.44, *p* =0.235	***F*(1, 46.9)=8.02, *p* =0.006** ^*^	***F*(1, 47)=4.63, *p* =0.036** ^*^
*Lang*	***F*(1, 1,684)=57.02, *p* <0.001** ^**^	*F*(1, 1665.2)=0.85, *p* =0.358	***F*(1, 2,505)=86.21, *p* <0.001** ^**^	***F*(1, 3426.8)=4.57, *p* =0.032** ^*^
*OS*	*F*(1, 155.3)=2.65, *p* =0.105	*F*(1, 158.3)=2.85, *p* =0.093	*F*(1, 157.2)=0.33, *p* =0.565	*F*(1, 161.3)=3.68, *p* =0.056
*PS*	***F*(1, 155.3)=20.91, *p* <0.001** ^**^	***F*(1, 158.3)=14.18, *p* <0.001** ^**^	*F*(1, 157.2)=0.06, *p* =0.806	***F*(1, 161.3)=12.22, *p* <0.001** ^**^
Group*Lang	***F*(1, 8613.1)=30.34, *p* <0.001** ^**^	*F*(1, 9079.3)=1.81, *p* =0.179	***F*(1, 7663.3)=13.66, *p* <0.001** ^**^	*F*(1, 9069.8)=2.23, *p* =0.136
Group*OS	*F*(1, 8613.4)=0.01, *p* =0.898	***F*(1, 9079.7)=6.94, *p* =0.008** ^*^	*F*(1, 7662.1)=2.15, *p* =0.143	***F*(1, 9069.5)=14.58, *p* <0.001** ^**^
Group*PS	*F*(1, 8613.6)=2.23, *p* =0.135	*F*(1, 9080.2)=0.70, *p* =0.403	*F*(1, 7662.5)=3.19, *p* =0.074	*F*(1, 9069.5)=0.01, *p* =0.905
Lang*OS	***F*(1, 1683.6)=11.30, *p* <0.001** ^**^	*F*(1, 1665.2)=1.04, *p* =0.309	***F*(1, 2502.8)=10.47, *p* =001** ^*^	*F*(1, 3426.6)=0.09, *p* =0.759
Lang*PS	*F*(1, 1683.5)=2.86, *p* =0.091	*F*(1, 1665.1)=0.84, *p* =0.359	*F*(1, 2504.2)=0.42, *p* =0.516	*F*(1, 3426.5)=0.16, *p* =0.686
OS*PS	*F*(1, 155.3)=1.07, *p* =0.302	*F*(1, 158.3)=3.01, *p* =0.085	*F*(1, 157.2)=0.24, *p* =0.622	*F*(1, 161.3)=0.611, *p* =0.436
Group*Lang*OS	*F*(1, 8,613)=0.28, *p* =0.597	*F*(1, 9079.5)=0.95, *p* =0.329	*F*(1, 7662.2)=2.49, *p* =0.114	***F*(1, 9069.5)=10.56, *p* =0.001** ^*^
Group*Lang*PS	***F*(1, 8,613)=4.61, *p* =0.032** ^*^	*F*(1, 9,079)=0.49, *p* =0.485	***F*(1, 7662.4)=6.15, *p* =0.013** ^*^	*F*(1, 9069.9)=0.37, *p* =0.539
Group*OS*PS	*F*(1, 8613.3)=0.09, *p* =0.755	*F*(1, 9079.1)=0.01, *p* =0.963	*F*(1, 7662.4)=0.01, *p* =0.941	*F*(1, 9069.7)=0.71, *p* =0.399
Lang*OS*PS	*F*(1, 1683.6)=1.85, *p* =0.173	***F*(1, 1665.2)=6.36, *p* =0.012** ^*^	*F*(1, 2504.1)=0.01, *p* =0.965	***F*(1, 3426.6)=5.58, *p* =0.018** ^*^
Group*Lang*OS*PS	*F*(1, 8,613)=0.23, *p* =0.631	*F*(1, 9079.9)=0.01, *p* =0.936	*F*(1, 7662.2)=0.02, *p* =0.889	*F*(1, 9069.7)=0.31, *p* =0.578

Lang, language; OS, orthographic similarity; and PS, phonological similarity.

* *p<* 0.05;

** *p<* 0.01. Significant effects are bolded

#### First Key Performance

##### Latency

Regarding the latency (RTs) of the *first key*, the main effect of Language was significant. The responses in English (mean=658ms) were faster than in Spanish (mean=763ms). The main effect of PS was also significant. Cognates with high PS (mean=648ms) were typed faster than of cognates with low PS (mean=773ms).

The interaction between Group and Language was significant. The differences between Spanish and English were significant in both groups [LBs: *t* (8612.6)=−9.23, *SE*=16.1, *p*<0.001; HSs: *t* (8610.3)=−3.86, SE=15.9, *p*<0.001], but the magnitude of the differences was greater in the LBs (Spanish: 772 – English: 623=149ms) than in the HSs (Spanish 754 – English: 692=22ms). The interaction between Language and OS was also significant, indicating that in the English block, there were no significant differences between cognates with high OS (mean=659ms) and cognates with low OS [mean=657ms; *t* (1680.9)=0.07, *SE*=30.7, *p*=0.945], whereas in the Spanish block, these differences were significant (mean of cognates with high OS=717ms; mean of cognates with low OS=808ms; *t* (1681.7)=− 2.97, *SE*=30.7, *p*=0.002).

The three-way interaction between Group, Language, and PS was also significant. In order to explore this interaction, we performed a specific model (Language^*^ PS) for each group separately. Here, we wanted to examine the interaction between Language and PS separately for the LBs and the HSs in order to examine the PS effect in each language, across the two language background profiles. The analysis performed in the LBs indicated a main effect of Language [*F* (1, 1797.2)=51.01, *p*<0.001] and PS [*F* (1, 149.28)=20.73, *p*=0.003]. In addition, the Language × PS interaction was significant, *F* (1, 1796.97)=9.75, *p*=0.002. During the English version of the task, cognates with high PS (mean=591ms) were typed faster than of cognates with low PS (mean=672ms), *t* (1793.45)=−2.85, *SE*=34.6, *p*=0.043. During the Spanish version of the task, cognates with high PS (mean=662ms) were also typed faster than of cognates with low PS (mean=853ms), *t* (1789.7)=−5.51, *SE*=34.7, *p*<0.001. Although in both languages there were differences between conditions, the magnitude of the differences was greater in Spanish (191ms) than in English (81ms). In HSs, there was a main effect of Language, *F* (1, 2839.1)=9.68, *p*=0.002. The responses in English (mean=631ms) were faster than in Spanish (mean=757ms). The main effect of PS was also significant, *F* (1, 148.85)=18.44, *p*<0.001. Thus, cognates with high PS (mean=627ms) were typed faster than cognates with low PS (mean=762ms) but the Language × PS interaction was not significant [*F* (1, 2838.04)=1.49, *p*=0.22]. No other interactions were significant.

##### Accuracy

Regarding the accuracy (ACC) of the *first key*, the main effect of PS was significant. The accuracy of cognates with high PS (mean=0.977) was higher than of cognates with low PS (mean=0.922).

The Group × OS interaction was also significant. In the LBs, the difference between cognates with high OS (mean=0.929) and cognates with low OS (mean=0.964) was significant [*t* (9076.8)=−2.36, SE=0.015, *p*=0.018], but in the HSs were not significant [O + mean=0.946; O− mean=0.959; *t* (9075.4)=−0.882, SE=0.015, *p*=0.377].

The three-way interaction between Language, OS, and PS was also significant. In order to explore the interaction, we performed a specific model (OS^*^PS) for each language separately. Here, we wanted to examined the interaction between OS and PS separately for each language in order to examine the interplay of OS and PS effect in each language. In the analysis performed in the English block, there was a main effect of PS, *F* (1, 92.01)=6.74, *p*=0.011. The accuracy of cognates with high PS (mean=0.984) was higher than of cognates with low PS (mean=0.928). However, the main effect of OS *F* (1, 92.01)=1.10, *p*=0.296 and the OS × PS interaction *F* (1, 92.01)=0.20, *p*=0.65 were not significant. The analysis performed in the Spanish block indicated that there was no main effect of OS, *F* (1, 100.98)=0.1.941, *p*=0.166, but the main effect for PS *F* (1, 100.99)=7.84, *p*=0.006 and OS × PS interaction were significant *F* (1, 100.89)=3.93, *p*=0.048. This interaction indicated that in the high OS condition, there were differences between the P+ (mean=0.977) and P− (mean=0.873) conditions; *t* (100.34)=3.622, *SE*=0.028, *p*<0.001. However, the difference between P+ (mean=0.965) and P− (mean=0.946) in the low OS condition was not significant; *t* (100.48)=0.573, *SE*=0.033, *p*=0.567.

#### Rest of the Word Performance

##### Latency

Regarding the latency (RTs) for the *rest of word*, the main effects of Group and Language were significant, indicating that the responses in the LBs (mean=1246ms) were faster than in the HSs (mean=1438ms) and that the responses in English (mean=1221ms) were faster than those in Spanish (mean=1463ms).

The interaction between Group and Language was significant. Differences between Spanish and English were significant for both groups [LBs: *t* (7661.8)=−6.630, *SE*=29.2, *p*<0.001; HSs: *t* (7662.8)=−9.944, SE=29.2, *p*<0.001], but the magnitude of the differences was greater in the HSs (Spanish: 1583 – English: 1293=290ms) than in the LBs (Spanish 1,343 – English: 1150=193ms). The interaction between Language and OS was also significant, indicating that the cognates with high OS were typed faster than cognates with low OS, but only in Spanish [O+ mean=1403ms; O− mean=1523ms; *t* (2489.3)=−2.963, *SE*=80.1, *p*=0.038]. In English, there were no differences [O+ mean=1245ms; O− mean=1195ms; *t* (2493.5)=1.012, *SE*=81.4, *p*=0.472].

The three-way interaction between Group, Language, and PS was also significant. To explore this interaction, we performed a specific model (Language^*^PS) for each group separately. Here, we wanted to examined the interaction between language and PS separately for the LBs and the HSs in order to examine the PS effect in each language, across the two language background profiles. The analysis in the LBs indicated that there was a main effect of Language [*F* (1, 3003.7)=46.22, *p*<0.001], indicating that the responses in English (mean=1,163ms) were faster than those in Spanish (mean=1,331ms). The main effect of PS [*F* (1, 152.46)=0.08, *p*=0.772] and the Language × PS interaction [*F* (1, 3003.22) =0.02, *p*=0.874] were not significant. The analysis for the HSs showed a main effect of Language [*F* (1, 3003.7)=66.17, *p*<0.001], indicating that the responses in English (mean=1316ms) were faster than those in Spanish (mean=1553ms). However, the main effect of PS was not significant [*F* (1, 155.43)=0.36, *p*=0.545]. The Language x PS interaction was significant [*F* (1, 3361.4.22)=3.74, *p*=0.039], so that in the English block, the difference between cognates with high PS (mean=1314ms) and cognates with low PS (mean=1319ms) was not significant, *t* (3002.3)=−0.069, *SE*=78.2, *p*=0.999, whereas in the Spanish block, the difference between cognates with high PS (mean=1512ms) and cognates with low PS (mean=1615ms) was significant, *t* (7664.1)=−2.55, *SE*=77.9, *p*=0.019.

##### Accuracy

Regarding the ACC of the *rest of word*, the main effect of Group was significant, with higher accuracy for the LBs (mean=0.858) than for the HSs (mean=0.833). The main effect of Language was also significant, indicating higher ACC in Spanish (mean=0.858) than in English (0.833). The main effect of PS was also significant. The accuracy of cognates with high PS (mean=0.899) was higher than of cognates with low PS (mean=0.792).

The Group × OS interaction was significant, so that in the LBs, the difference between cognates with high OS (mean=0.875) and cognates with low OS (mean=0.841) was not significant [*t* (9067.7)=1.097, *SE*=0.034, *p*=0.273], whereas this difference was significant for the HSs [O+ mean=0.874; O− mean=0.791; *t* (9068.5)=2.661, *SE*=0.031, *p*=0.008].

The three-way interaction between Group, Language, and OS was significant. To explore this interaction, we performed a specific model (Language × OS) for each group separately. The analysis in the LBs indicated that there was a main effect of Language, [*F* (1, 1664.11)=5.05, *p*=0.024], indicating higher ACC in Spanish (mean=0.870), than in English (0.835). The main effect of OS [*F* (1, 160.24)=2.21, *p*=0.138] and the Language × OS interaction were not significant [*F* (1, 1664.08)=1.28, *p*=0.257]. The analysis for the HSs showed that the main effect of Language was not significant [*F* (1, 1740.22)=0.08, *p*=0.768]; however, the main effect of OS [*F* (1, 161.08)=6.81, *p*=0.009] and Language × OS interaction were significant [*F* (1,1740.08)=3.77, *p*=0.05]. The interaction indicated that in the English block, the difference between cognates with high OS (mean=0.851) and cognates with low OS (mean=0.796) was not significant; [*t* (1738.7) =1.499, *SE*=0.037, *p*=0.438], whereas in the Spanish block, this difference was significant [O+ mean=0.887; O− mean=0.769; *t* (1736.5)=3.198, *SE*=0.037, *p*=0.008].

The three-way interaction between Language, OS, and PS was significant. We explored this interaction by a specific model (OS × PS) for each language separately. The analysis in the English block showed no main effect of OS, *F* (1,103.69)=0.62, *p*=0.431, but the main effect of PS *F* (1,103.69)=10.84, *p*=0.001 and the OS × PS interaction were significant, *F* (1,103.69)=4.32, *p*=0.023. This interaction indicated that for the high OS condition, there were no differences between the high PS (mean=0.919) and the low PS (mean=0.840) conditions; *t* (102.6)=1.375, SE=0.057, *p*=0.515. However, there were differences between high PS (mean=0.917) and low PS (mean=0.740) in the low OS condition; *t* (101.7)=3.163, SE=0.066, *p*=0.008. The analysis in the Spanish block showed main effects of OS, [*F* (1,102.85)=6.20, *p*=0.014], with higher accuracy for cognates with high OS (mean=0.868) than for cognates with low OS (mean=0.799). The main effect of PS was also significant, *F* (1,102.85)=6.78, *p*=0.012, so that accuracy in cognates with high PS (mean=0.871) was higher than in cognates with low PS (mean=0.777). The OS × PS interaction was not significant, *F* (1, 102.85)=0.336, *p*=0.563. No other effects or interactions reached significance.

#### Summary of the OS and PS Analysis

The results indicated that for the *first key*, the effect of the PS was present in the two languages and for the two groups (i.e., participants processed high PS cognates faster than low PS cognates), although PS effects were stronger for the HSs than LBs. In LBs, the PS effect was stronger in Spanish than in English. OS had an effect in the Spanish block (i.e., participants processed high OS cognates faster than low OS cognates) but this effect interacted with PS. That is, the difference between P+ and P− conditions was significant only for the high OS condition. In English, there was no effect of OS.

For the *rest of word*, the effect of PS in reaction time was restricted to Spanish in the HSs. However, in Spanish, it was present for accuracy (cognates with high PS had a better performance than cognates with low PS), but in English depended on OS (in cognates with high OS, there were no differences between P+ and P−. However, in low OS cognates, the accuracy was higher for P+ than P− cognates). Regarding the accuracy, the effect of OS depended on group and language, so that for the HSs, the effect appeared in Spanish, but not in English, whereas in the LBs, the effect was not evident. OS tended to be more influential for typing the rest of the word than the first key (which was more influenced by PS).

## Discussion

The main goal of this study was to investigate language co-activation and the role of cognate status during bilingual writing using a writing to dictation task. More specifically, we investigated the relative contributions of the profile of participants’ language backgrounds by testing two bilingual populations: LBs (L1: English; L2: Spanish) and HSs (Majority language: English; Minority language: Spanish) which were both immersed in an English dominant context but differed in the level of formal literacy received in Spanish. The main goal was to analyze performance during typing of cognate and non-cognate words and examine how different degrees of orthographic similarity (OS) and phonological similarity (PS) in cognates affected writing times and accuracy. Importantly, from a theoretical standpoint, it is not completely clear how non-identical but similar cognates are lexically represented, what the role of orthographic and phonological similarity is in shaping these representations, especially when bilingualism is modulated by more or less exposure to formal education in one language. Moreover, previous experiments on cognate similarity have used reading tasks with visual presentations which may have obscured the role of phonological similarity. Critically, here, we use a writing to dictation task in which words were orally presented but orthographically implemented, therefore providing a tool to unveil the role of both phonological and orthographic similarities. In addition, and very key to this study, the use of writing could also unveil possible differences in the nature of language co-activation for bilinguals with different language experiences. In the following subsections, we will discuss the reported results to examine the influence of cognate status, the impact of OS and PS in language co-activation, and the diversity of language and learning backgrounds on the current task.

### The Consequences of Co-activation in Writing to Dictation: The Overall Effect of Cognate Status

The results of our experiment shed some light on the nature of cognate effects during a writing to dictation task. Previous studies have shown that cognates are “special” because they share more semantic, orthographic, and phonological characteristics between languages than non-cognates (Voga and Grainger, 2007). Cognate facilitation effects have been widely reported in bilinguals and reflect language co-activation in reading, visual word recognition (e.g., Lemhöfer and Dijkstra, 2004; Dijkstra et al., 2010; Peeters et al., 2013), and in translation (Muscalu and Smiley, 2018). In the present experiment, cognate effects were also modulated by the language experience of the bilingual and the language in which the writing task was performed. More specifically, cognate facilitation was only present in HSs while processing in the minority language (Spanish), providing evidence of co-activation with the majority language (English). However, the results demonstrated an unexpected cognate interference effect in English (L1/majority language) with cognates being less accurate and slower than non-cognates in both groups.

Although cognate interference is not a common finding, some previous studies have found a similar effect (Schwartz et al., 2007; Dijkstra et al., 2010; Comesaña et al., 2012; Muscalu and Smiley, 2018). Critically, in all of them, non-identical cognates were included as experimental material suggesting that the degree of OS and PS in cognates may have an important impact during word processing. The BAST (Tainturier, 2019) has proposed that the strength of co-activation is mediated by the degree of OS and PS between the two languages, so the relative proportion of high and low similarity cognates can modulate the resulting facilitation vs. interference effects. Importantly, in the present study, cognates with high orthographic and phonological similarity (O+P+) were intermixed with cognates with low OS or PS (O−P+ and O+P−) and cognates with low OS and PS (O−P−). The fact that low similarity cognates represented one-third of the cognate stimuli might have masked the expected cognate facilitation effect. Thus, cognates are generally expected to produce co-activation of the two languages, and in turn facilitation, but the salient change in the code/representation (orthographic or phonological) of non-identical cognates may have produced competition and impaired their processing. At this point, competition between the two language representations would trigger lateral inhibition in order to reduce interference and select the appropriate representation (for a similar interpretation see Comesaña et al., 2012). Because non-cognates produce much weaker between language co-activation than cognate words, competition between representations would also be weaker for non-cognates relative to cognates (even for low similarity cognates). The role of inhibition when selecting among lexical competitors has also been proposed by others (Borragan et al., 2018; Broersma et al., 2016; Filippi et al., 2014). In line with this interpretation, previous research has found larger error monitoring effects and higher recruitment of brain regions dedicated to control while processing non-identical cognates relative to control words (Declerck et al., 2017; Peeters et al., 2019).

In addition, our results showed that the interference effect was found in the L1/majority language in both LBs and HSs, replicating previous production studies which showed a reversed dominance effect, exemplified by more intrusion errors in the dominant language (Gollan et al., 2014; Gollan and Goldrick, 2016; Li and Gollan, 2018). In this direction, some studies have pointed out that language processing in the L1/majority language could be largely mediated by an automatic process of orthography to phonology conversions, while processing in the L2 is more attentionally demanding (Plat et al., 2018). We propose that the manipulated similarities and differences in phonology and orthography in the current study might have directly affected the phonology to orthography conversion (POC). Since the L1/majority language is mediated by automatic processes, it is easier to observe interference effects. On the contrary, during L2/minority language, processing is more demanding, and therefore, the interference effect is reduced. The fact that interference occurs for HSs in the majority language (English) even though Spanish is their L1 may suggest that the regulatory processes are dependent on language experience and proficiency.

### The Nature of Language Co-activation: The Role of PS and OS

The strength of language co-activation is mediated by the degree of orthographic and phonological similarity between languages (Tainturier, 2019). Nevertheless, orthographic processing has been the focus of most studies (e.g., Van Kesteren et al., 2012; Peeters et al., 2013; Casaponsa et al., 2014; Hoversten et al., 2017; Muscalu and Smiley, 2018), reporting in a general larger cognate facilitation effects with greater OS (Dijkstra et al., 2010). Crucially, cognates can also vary in the degree of phonological similarity (PS) across languages. However, the role of PS and the interaction of PS with OS have received little to no attention. Very few studies have explored the interplay of PS and OS during word processing and most have relied on a reading task in which orthographic processing is imperative (Schwartz et al., 2007; Comesaña et al., 2012). For example, previous studies have demonstrated that the positive effect of PS (i.e., faster RTs for cognates with high PS than cognates with low PS) was mediated by the OS (Schwartz et al., 2007; Comesaña et al., 2012). In those studies, the PS effects only emerged in high OS conditions (i.e., the response in O+P+ condition was faster than the responses in O+P− condition). However, there were no differences between high and low PS in cognates with low OS (there were no differences between O – P+ and O−P−). In other words, if common orthographic L1/L2 nodes map into different phonological L1/L2 nodes, it can create confusion, slowing down the processing of the word (Doctor and Klein, 1992; Dijkstra et al., 1999; Dijkstra and Van Heuven, 2002; Schwartz et al., 2007).

The absence of PS effects in low OS conditions reported in previous studies has been explained by the orthographic autonomy hypothesis which proposes that written production is not dependent on spoken production and therefore not dependent on phonological information (Rapp and Caramazza, 1997). In reading, orthographic retrieval is mandatory, and the co-activation of language nodes would be mediated by OS. In addition, in the O− condition, the co-activated representations compete for selection, and inhibition would be triggered to achieve successful processing (in the Comesaña and colleagues’ and in the Schwartz and colleagues’ studies). This first orthographic filter would reduce the spread of activation to phonology (see Figure 1 in which the arrows on the left represent the reduction of spread in the O− condition). As mentioned, phonological processing in reading is delayed with respect to orthographic processing because the stimuli are visually presented and mapping of orthography to phonology only occurs after orthographic analyses have taken place. However, writing production paradigms, and especially writing to dictation tasks, can be key to study the role of phonology because these tasks involve phonological input and orthographically oriented responses, such that phonological processing is mandatory (obligatory phonological mediation hypothesis; Geschwind, 1969).

Contrary to previous studies, the results of our writing to dictation task showed a general PS effect in the first key latency and accuracy in most conditions of the experiment. In the first key latency analysis, the PS effect (faster RTs for cognates with high PS than cognates with low PS) was present for both groups (LBs and HSs) in English (L1/majority language) and Spanish (L2/minority language), suggesting primacy of phonological processing facilitating the access to the lexical representations of the words. The first filter would therefore be phonological, so in the low OS condition, phonological information would continue to be processed, because the first filter, in this case, did not reduce the spread of activation to phonology (see Figure 1, specifically see the arrows on the far right).

In contrast, PS effects for the rest of the word, although present in accuracy, were not present in LBs and it interacted with OS in HSs (in Spanish). This pattern suggests that the role of phonology is smaller as the time course progresses and the influence of orthography gains relevance. The fact that the OS effect was more consistently found in Spanish than in English in the rest of the word analyses suggests that the way the words are processed in each language could be different (i.e., after the first key). Performance on the rest of the word in the writing task has been attributed to sublexical processing (Muscalu and Smiley, 2018; Iniesta et al., 2021). Dual-route theories of reading propose that transparent orthographies, such as Spanish, rely on phoneme to grapheme processing, contrary to deeper languages, such as English, which uses direct access to lexical representations (orthographic depth hypothesis; Frost, 1994, 2012). So, the OS, which is a sublexical characteristic, would more directly affect sublexical processing (the POC system) than lexical processing explaining the greater role of OS in Spanish.

In sum, differences in the time course of orthographic and phonological activation during reading vs. writing to dictation tasks explain the differences in the impact of OS and PS. The bilingual interactive activation BIA+ model (Dijkstra and Van Heuven, 2002) introduces the “temporal delay assumption” to explain that under some conditions, cross-linguistic phonological, orthographic, and/or semantic effects may be absent due to task demands. Reading requires orthographic activation prior to phonological activation, and therefore, the late phonological activation would not affect response times (Brysbaert et al., 2002). However, during a writing to dictation task, the phonological processing precedes activation of orthographic information, and therefore, the phonology may directly impact the performance. The fact that phonological processing occurs early in writing to dictation explain the generalized PS effects in all experimental conditions (faster responses for cognates with high PS than for cognates with low PS).

Following previous studies, we decided to use OS and PS as dichotomous variables to directly compare reading and writing to dictation (Schwartz et al., 2007; Comesaña et al., 2012). However, the threshold used to classify cognates as high or low similarity is somewhat arbitrary, and future research in this field should consider OS and PS as continuous variables.

### The Role of the Learning Environment in Language Co-activation

In our experiment, we included two groups of bilinguals: LBs and HSs. We hypothesized that the relationship between the L1 and the L2 could be influenced by their linguistic learning background (Kroll et al., 2006; Dijkstra et al., 2010). More specifically, differences in literacy and exposure to writing and reading between the two groups might modulate the co-activation effects and the relative roles of OS and PS in L1 and L2 processing. The two groups did not differ in the LEAP-Q measures for English (L1/majority language): There were no differences in the years of schooling in an English context, nor in their self-assessed language skills for speaking, understanding, and reading, nor in their reading contribution to learning measure which reflects L1 formal learning and regulates learning at school (Iniesta et al., 2021). For Spanish, however (L2/minority language), the LEAP-Q highlighted significant differences in the self-assessed skills in speaking and understanding with HSs scoring higher than LBs. Critically, in skills that were more related to formal language use, like reading, there were no group differences, and additionally, HSs showed a lower score for the score reading contribution to learning. In addition, and in accordance with previous studies (Elola and Mikulski, 2016), scores in the Spanish tests showed worse performance for HSs than LBs (PROESC and TECLE). Hence, even though the years of exposure to Spanish were greater in the HSs, they showed more orthographic difficulties in Spanish than the LBs, presumably due to the fact that their input during learning was mainly phonological, resulting in a less accumulated literacy practice (see the weaker links hypothesis; Gollan et al., 2008).

In the same direction of PROESC and TECLE, the HSs showed worse performance in the writing to dictation task (relative to LBs), specifically in the latency of the rest of word performance. This suggests that the HSs might have greater difficulties in sublexical processing, where the orthographic form retrieval is especially important. In addition, analysis of the RTs showed an interaction between Group and Language. This interaction indicated that both groups were faster in English than Spanish, but the magnitude of the difference was greater for the LBs than for the HSs when looking at the first key performance (lexical access), and the magnitude of the difference was greater for the HSs than the LBs when considering the rest of the word performance (sublexical processing). Again, this pattern suggests that the HSs might have more difficulties retrieving the word form in both languages (English and Spanish) although these difficulties become more evident during writing production in the minority language, presumably due to less accumulated practice as a result of their learning background (see also Gollan et al., 2008).

Regarding the OS and PS between languages, there were subtle differences between groups. In the RTs analysis of the first key (lexical) latency, the results showed a Group × Language × PS interaction. Even though there were significant differences between high PS and low PS in both groups and both languages (Spanish and English), the magnitude of the difference was higher in Spanish in the LBs (cognates with high PS were typed faster than cognates with low PS). A possible interpretation of this effect is that when LBs type the first key in Spanish, the English phonology is more co-activated than when they are typing in English and Spanish is co-activated. In contrast, for HSs, there were no magnitude differences between languages for phonology. There were no accuracy differences while processing O+ and O− cognates for the first key, suggesting that for HSs the sensitivity to the OS is reduced in both languages. This pattern supports previous studies that show phonological advantages for HSs relative to LBs (Chang et al., 2011; Gor, 2014), but also orthographic disadvantages (Elola and Mikulski, 2016).

In sum, these results add to the current literature on bilingual language co-activation by demonstrating that the language learning environment, especially formal exposure to reading and writing in a given language, can not only modulate proficiency but also affect how the languages are co-activated and how they interact.

## Conclusion

The present study provides evidence that language co-activation during writing production in L1 and L2 is modulated by OS, but also, and more important, by PS across languages. Writing to dictation involves phonology from the very early processing stages so that PS contributes to facilitating access to the lexical representation of the words. Hence, contrary to previous studies on reading, the PS effects were very pervasive during lexical access (first key latency, [i.e., participants process cognates with high PS faster than cognates with low PS]), although they showed modulation with orthography during the implementation of writing while typing the rest of the word (sublexical processing). In contrast, the effect of OS was not extensively evident during lexical access (first key), and it had a more important role during the sublexical processing (rest of the word). In addition, the results provide evidence about the impact of literacy differences for orthographic and phonological co-activation during writing production (in this case, the acquisition of the Spanish L2/minority language).

To conclude, the interplay of OS and PS underlying cross-linguistic influence in bilinguals seems to be dependent on the relative order in which orthographic and phonological processing occur, and this pattern can be modulated by the task that bilinguals are performing and by the language learning environment of the bilinguals. Commonly, bilingual competence is conceptualized as a continuum. In this continuum, the study of HSs is especially important because it allows for an exploration of how different cultural, linguistic, and educational contexts influence language learning and the relationship between languages.

## Data Availability Statement

The raw data supporting the conclusions of this article will be made available by the authors, without undue reservation.

## Ethics Statement

The studies involving human participants were reviewed and approved by the University of Florida Institutional Review Board (IRB): protocol #2019–02427. The patients/participants provided their written informed consent to participate in this study.

## Author Contributions

AI, DP, and TB contributed the conception and design of the study. AI and ER organized the data collection. AI and DP performed the statistical analysis. AI wrote the first draft of the manuscript. All authors wrote sections of the manuscript and contributed to the manuscript revision, read, and approved the submitted version.

## Funding

The current research was completed and thanks to financial aid provided by the doctoral research grant FPU16/01748 to AI and grants from the Ministerio de Ciencia, Innovación y Universidades-Fondos Feder to TB (PGC2018-093786-B-I00) and DP (PCIN-2015-165-C02-01 and PSI2017-89324-C2-1-P) and from the Feder Andalucía to TB (A-CTS-111-UGR18 and P20.00107).

## Conflict of Interest

The authors declare that the research was conducted in the absence of any commercial or financial relationships that could be construed as a potential conflict of interest.

## Publisher’s Note

All claims expressed in this article are solely those of the authors and do not necessarily represent those of their affiliated organizations, or those of the publisher, the editors and the reviewers. Any product that may be evaluated in this article, or claim that may be made by its manufacturer, is not guaranteed or endorsed by the publisher.
